# Analysis of Avian Hepatitis E Virus from Chickens, China

**DOI:** 10.3201/eid1609.100626

**Published:** 2010-09

**Authors:** Qin Zhao, En Min Zhou, Shi Wei Dong, Hong Kai Qiu, Lu Zhang, Shou Bin Hu, Fei Fei Zhao, Shi Jin Jiang, Ya Ni Sun

**Affiliations:** Author affiliation: Shandong Agricultural University, Shandong, People’s Republic of China

**Keywords:** Avian hepatitis E virus, HEV, sequence, analysis, viruses, China, dispatch

## Abstract

Avian hepatitis E virus (HEV) has been identified in chickens; however, only 4 complete or near-complete genomic sequences have been reported. We found that the near-complete genomic sequence of avian HEV in chickens from China shared the highest identity (98.3%) with avian HEV from Europe and belonged to avian HEV genotype 3.

Hepatitis E virus (HEV) is a nonenveloped, positive-sense, single-stranded RNA virus. It has 3 open reading frames (ORFs) and a genome size of 7.2 kb (*1*). So far, HEV strains are classified into 4 major genotypes, and genotypes 3 and 4 are probably zoonotic.

Avian HEVs have been identified from chickens with big liver syndrome and hepatitis–splenomegaly syndrome. Each syndrome mainly causes increased deaths, reduced egg production, and enlarged liver and spleen (*2*); hepatitis–splenomegaly syndrome also causes accumulation of bloody fluid in the abdomen and vasculitis and amyloidosis in the liver (*3*,*4*). Molecular epidemiologic investigations have shown that avian HEV infection in chickens is endemic to the United States and Spain (*5**,**6*). Because propagating avian HEV in cell culture or embryonated eggs is difficult (*2**,**3*), avian HEV is primarily detected by reverse transcription–PCR (RT-PCR). However, only 4 complete or near-complete genomic sequences have been reported to GenBank (*7**–**9*). We identified and analyzed the near-complete genomic sequence of avian HEV in a chicken flock from the People’s Republic of China.

## The Study

In May 2009, hepatitis–splenomegaly syndrome affected a flock of 37-week-old broiler breeder hens in Shandong, China. This flock had a history of decreased egg production. Affected chickens had regressive ovaries, extensive necrosis and hemorrhage of the liver, and enlarged liver and spleen. Antibodies against avian HEV ORF2 were detected in 80 of 94 serum samples from the same chicken flock, according to ELISA (*5**,**10*) with the truncated ORF2 protein used by Guo et al (*10*) and chicken serum diluted 1:100 in 0.5% Tween-20 phosphate-buffered saline containing 2.5% nonfat dry milk and 10% *Escherichia coli* lysate. On the basis of previous results, we used a cutoff optical density of 0.43 (*11*). Using a published method (*12*), we detected an avian HEV ORF2 RNA gene with 242 bp in 7 of 10 fecal and 5 of 8 bile samples.

From the bile samples that were positive for the avian HEV ORF2 gene, we used nested RT-PCR with 5 overlapping fragments to amplify the near-complete genomic sequence of avian HEV. Primers were designed on the basis of the other 4 avian HEV near-complete sequences in GenBank (Table 1). The RT-PCR conditions and reaction mixture were designed according to the SuperScript II One-Step RT-PCR System instructions (Invitrogen, Carlsbad, CA, USA). To identify the extreme 3′ genomic sequence, we used a modified RACE (3′ rapid amplification of cDNA ends) technique. The sense primer F5 (Table 1) was chosen from the ORF2 region, and the antisense primers included a commercially available anchored adaptor primer and an amplification primer (Invitrogen). Using inner PCR primers, we sequenced the PCR products of 5 fragments in both directions (Table 1); the sequence data were collected by an ABI3730 Genetic Analyzer (JinSiTe Biotech Co., Nanjing, China).

**Table 1:** Primers used for PCR amplification of the China avian hepatitis E virus genome

Primer*	Sequence, 5′ → 3′†	Position, nt‡
F1-1	CCATGCCAGGGTAAGAATG	9–27
R1-1	AAAACAGCAAGGACCTCC	1872–1889
F1-2	CCAGGGTAAGAATGGACG	14–31
R1-2	TAATCCAGGTGGCGAGC	1308–1324
F2-1	CACTGTGGGTAACATTGTGGC	1071–1091
R2-1	GTTCGACTGCTTAGCCACCTG	2935–2955
F2-2	AGGCGGAACACGCACAGCA	1214–1232
R2-2	TCGTCCACAATGACCCTGC	2624–2642
F3-1	GGCTGTGTGGCATGTTCCA	1985–2003
R3-1	GGTAAAGAGCCACCATCCAAT	4010–4030
F3-2	CCGTGATGGTGACTTGTTGGTTGT	2262–2285
R3-2	GGCACATCTCCGCATACTC	3586–3604
F4-1	CCCTTCAACATTGGAGTATGC	3573–3593
R4-1	ATCTGGTACCGTGCGAGT	4899–4916
F4-2	ACATTGGAGTATGCGGAGATG	3580–3600
R4-2	TTGAGCGCTCCACTGGGCT	4820–4838
F5	GACAATTCAGCCCAGTGGA G	4809–4828
AUAP§	GACTCGAGTCGACATCG A	Nonviral
AP§	GACTCGAGTCGACATCGA (T)_17_	Nonviral

*Primers F1-1 to R1-2, F2-1 to R2-2, F3-1 to R3-2, and F4-1 to R4-2 were used to amplify the first, second, third, and fourth fragment of the near-complete avian hepatitis E virus (HEV) genome. Primers F5, amplification primer (AUAP), and adapter primer (AP) were used to amplify the extreme 3′ genomic sequence. Primers R1-1, R2-1, R3-1, R4-1, and AP are also reverse transcription primers. †Sequences of primers were designed according to the sequences of 4 other known avian HEV strains. ‡Positions of primers located in the complete genome are shown according to the Europe avian HEV isolate. §Commercial primer (Invitrogen, Carlsbad, CA, USA) of nonviral origin.

We assembled the near-complete genome of avian HEV, which was 6,660 nt long including the 3′ poly A tail, by using 5 overlapping fragments sequences and Lasergene 7.0 EditSeq computer programs (DNAStar, Madison, WI, USA) and designated it China avian HEV (CaHEV). CaHEV contained a complete ORF1 gene encoding a nonstructural protein of 1,522 aa, an ORF2 gene encoding a capsid protein of 606 aa, an ORF3 gene encoding a cytoskeleton-associated phosphoprotein of 87 aa, and a 3′ noncoding region of 121 nt. The sequences of CaHEV were deposited into GenBank under accession no. GU954430.

The near-complete genomic and different region sequence analyses performed by using ClustalW (www.clustal.org) and Lasergene 7.0 MegAlign software indicated that CaHEV shared the highest identity (98.3%) with European avian HEV isolate (EaHEV) and 82.0%–82.6% with 3 other avian HEV isolates (Table 2). Moreover, compared with the different regions of 4 other avian HEV strains, the ORF1 gene of CaHEV shared 80.7%–98.3% nt and 92.7%–98.8% aa sequence identities, the ORF2 gene shared 84.1%–98.5% nt and 98.3%–99.7% aa sequence identities, the ORF3 gene shared 93.9%–98.9% nt and 88.6%– 97.7% aa identities, and the 3′ noncoding region shared 78.9%–97.6% nt identities (Table 2).

**Table 2:** Percentage identities among avian HEV strains in nucleotide/amino acid sequences*

Sequence and strain	“Avirulent aHEV”	Prototype aHEV	AaHEV	EaHEV	CaHEV
Near-complete genome sequence					
“Avirulent aHEV”		90.1	82.7	82.9	82.6
Prototype aHEV			82.5	82.2	82.0
AaHEV				82.5	82.4
EaHEV					98.3
CaHEV					
ORF1					
“Avirulent aHEV”		89.6	82.1	81.8	81.7
Prototype aHEV	**97.4**		81.6	81.0	80.7
AaHEV	**93.9**	**93.7**		81.7	81.6
EaHEV	**92.9**	**93.0**	**93.1**		98.3
CaHEV	**92.7**	**92.8**	**93.0**	**98.8**	
ORF2					
“Avirulent aHEV”		90.7	84.5	84.0	84.1
Prototype aHEV	**99.0**		84.3	84.4	84.5
AaHEV	**98.5**	**98.8**		84.1	84.4
EaHEV	**98.2**	**98.7**	**98.8**		98.5
CaHEV	**98.3**	**99.0**	**98.8**	**99.7**	
ORF3					
“Avirulent aHEV”		97.0	95.4	93.6	93.9
Prototype aHEV	**99.0**		95.4	93.6	93.9
AaHEV	**94.3**	**96.6**		93.5	93.9
EaHEV	**88.6**	**88.6**	**92.0**		98.9
CaHEV	**88.6**	**88.6**	**92.0**	**97.7**	
3′ NCR					
“Avirulent aHEV”		92.8	82.8	88.6	89.4
Prototype aHEV			83.6	85.5	86.3
AaHEV				80.5	78.9
EaHEV					97.6
CaHEV					

*HEV, hepatitis E virus; ORF, open reading frame; NCR, noncoding region. **Boldface** indicates percentage identities of amino acid sequences. “Avirulent aHEV” and prototype aHEV are avian HEV isolates from the United States, GenBank accession nos. EF206691 and AM535004, respectively. AaHEV, EaHEV, and CaHEV are avian HEV isolates from Australia, Europe, and China, GenBank accession nos. AM943647, AM943646, and GU954430, respectively.

**Figure 1:**
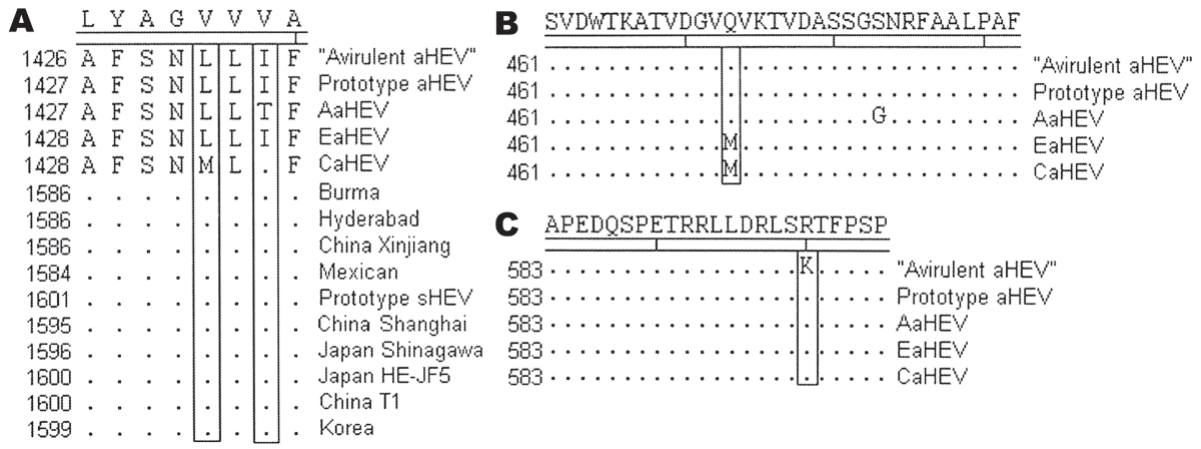
Amino acid sequence comparison of motif VII in the open reading frame (ORF) 1 RNA-dependent RNA polymerase (RdRp) region of avian, human, and swine hepatitis E viruses (HEVs) (A), antigenic domain II (B), and antigenic domain IV (C) in the ORF2 region of avian HEV. Residues that are conserved among avian HEV (aHEV) isolates are shown as the consensus above the sequences; residues that are conserved in the HEV strains are not shown. GenBank accession numbers of human and swine HEV (sHEV) strains are M73218 (Burma), AF076239 (Hyderabad), D11092 (China Xingjiang), M74506 (Mexico), AF082843 (prototype sHEV), FJ527832 (China Shanghai), AB291955 (Japan Shinagawa), AJ272108 (China T1), AB480825 (Japan HE-JF5), and FJ763142 (Korea). GenBank accession numbers of avian HEV strains are EF206691 (“avirulent aHEV” from the United States), AM535004 (prototype aHEV from the United States), AM943647 (aHEV from Australia [AaHEV]), AM943646 (aHEV from Europe [EaHEV]), and GU954430 (aHEV from China [CaHEV]). Boxes indicate mutations of CaHEV compared with different HEV strains.

ORF1 of CaHEV contained most mutations compared with prototype avian HEV (prototype aHEV); 5, 16, and 29 nonsilent mutations occurred in the methyltransferase, helicase, and RNA-dependent RNA polymerase (RdRp) functional domains, respectively (data not shown). However, only 2 mutations occurred in motif VII of RdRp domain (Figure 1, panel A), which contains 8 motifs responsible for virus replication (*13*). The 2 mutations in motif VII of the CaHEV RdRp domain are L(1432)M and I(1434)V. Australian avian HEV isolate (AaHEV) also has the mutation in the latter position and was a transition from I(1433) to T (Figure 1, panel A). This position is well conserved among mammalian HEV isolates by the presence of V, which is the same as CaHEV (Figure 1, panel A).

In the ORF2 region, 6 nonsilent mutations (C4R, R5G, G27S, T42A, T303V, and Q473M) were determined for CaHEV and compared with prototype aHEV. One mutation of Q(473)M, in the antigenic domain II, was seen in EaHEV and in CaHEV (Figure 1, panel B). Because this domain is unique to avian HEV, as predicted by Haqshenas et al. (*14*) and Guo et al. (*10*), this point mutation may change the antigenicity of the epitopes in domain II of the capsid protein. In antigenic domain IV, a mutation of R(600)K occurred in the “avirulent aHEV” compared with other 4 avian HEV strains, including CaHEV, from the sick chickens (Figure 1, panel C). This mutation may affect the virulence of avian HEV as speculated by Billam et al. (*8*) and Billic et al. (*9*). The 3 putative N-linked glycosylation sites (^255^NLS [1], ^510^NST [2], and ^522^NGS [3]) are shared between prototype aHEV and CaHEV (data not shown). However, the second site is ^510^NNT in “avirulent aHEV” and AaHEV strains and is eliminated in the EaHEV strain (*9*). In human and swine HEV strains, these sites are ^137^NLS (1), ^310^NLT (2), and ^562^NLS (3). Recently, the potential N-linked glycosylation in ORF2 was shown to prevent formation of infectious particles, but its role in other functions of HEV, e.g., virus virulence and cell tropism, remain to be elucidated (*15*). In the ORF3 gene, including only 83 aa, 10 nonsilent mutations were found compared with the prototype aHEV, and 9 mutations were the same as EaHEV (data not shown).

**Figure 2:**
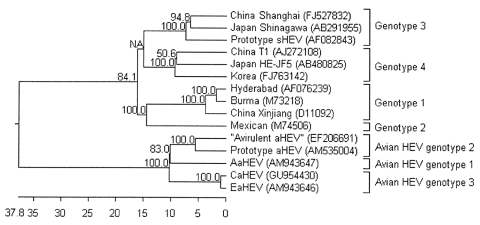
Phylogenetic trees based on the near-complete genomic sequences of avian hepatitis E virus (HEV) and 10 human and swine HEV isolates. GenBank accession numbers follow the name of HEV strains. The trees were constructed by the neighbor-joining method with 1,000 bootstrap replicates using Lasergene 7.0 (DNAStar, Madison, WI, USA). The length of each pair of branches represents the distance between sequence pairs; the units at the bottom of the tree indicate the number of substitution events.

Phylogenetic trees of the near full-length sequence of avian and mammalian HEV strains were constructed by using the neighbor-joining distance method and Lasergene 7.0 software. A bootstrap test of 1,000 replicates was used to evaluate the reliability of the groups. Avian HEV was segregated into a distinct branch separate from mammalian HEV; according to the genotype separation corresponding to their geographic origin suggested by Bilic et al. (*9*), CaHEV belongs to avian HEV genotype 3 (Figure 2).

## Conclusions

Avian HEV infection of a chicken flock in Shandong, China, was identified by detection of avian HEV ORF2 antibodies and viral RNA. A near-complete avian HEV genome from the flock was determined, and sequence analysis indicated that this avian HEV strain displayed the highest identity (98.3%) with EaHEV and belonged to avian HEV genotype 3.
